# The Impact of Pre‐Existing Psychiatric Disorders on Gastric Cancer Stage and Mortality in Older Adults

**DOI:** 10.1002/cam4.71622

**Published:** 2026-06-03

**Authors:** Josephine Soddano, Sophie Wagner, Ji Yoon Yoon, Jeong Yun Yang, Ling Chen, Yongmei Huang, Sheila D. Rustgi, Yoanna S. Pumpalova, Jennifer S. Ferris, Chin Hur

**Affiliations:** ^1^ Department of Medicine Columbia University Irving Medical Center New York New York USA; ^2^ Division of Gastroenterology Icahn School of Medicine at Mount Sinai New York New York USA; ^3^ Division of Digestive and Liver Diseases Columbia University Irving Medical Center New York New York USA; ^4^ Department of Obstetrics and Gynecology Columbia University Irving Medical Center New York New York USA

**Keywords:** cancer mortality, cancer stage, gastric cancer, psychiatric disorders, SEER‐Medicare

## Abstract

**Purpose:**

The association between pre‐existing psychiatric disorders (PD) and gastric cancer outcomes has not been thoroughly investigated. This study evaluated whether PD, particularly serious pre‐existing psychiatric disorders (SPD), is associated with differences in gastric cancer stage at diagnosis, overall mortality, and cancer‐specific mortality.

**Methods:**

We conducted a retrospective cohort study using SEER‐Medicare data (2000–2017) to identify patients aged ≥ 68 years diagnosed with gastric cancer. PD was defined as a Medicare claim for depressive disorder, anxiety disorder, adjustment disorder, psychotic disorder, bipolar disorder, or schizophrenia occurring 6–36 months before cancer diagnosis. SPD included claims for psychotic disorder, bipolar disorder, schizophrenia, or major depressive disorder (MDD). Multinomial logistic regression was used to assess stage at diagnosis. Cox proportional hazards and Fine‐Gray subdistribution hazard models were used to assess overall and cancer‐specific mortality, respectively.

**Results:**

Among 15,882 patients, 1883 (12%) had PD. Patients with PD differed significantly from those with no pre‐existing psychiatric disorders (NPD) in terms of demographics, comorbidities, and cancer characteristics. Compared with NPD patients, those with PD were more likely to be diagnosed with early (in situ/local) stage cancer yet had higher overall mortality (aHR: 1.17, 95% CI [1.11, 1.23]) and cancer‐specific mortality (aSHR: 1.13, 95% CI [1.07, 1.21]). Patients with SPD also had higher risks of overall (aHR: 1.33, 95% CI [1.22, 1.45]) and cancer‐specific mortality (aSHR: 1.25, 95% CI [1.14, 1.38]).

**Conclusion:**

Our findings highlight critical differences in gastric cancer outcomes among patients with PD and SPD. Despite being more likely to be diagnosed with early‐stage cancer, these patients experienced worse survival.

## Graphical abstract

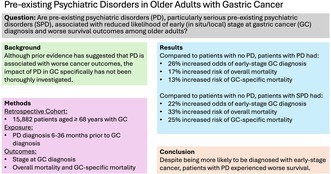

## 1 Introduction

Gastric cancer remains a significant global public health concern, ranking as the fifth most commonly diagnosed cancer and the fourth leading cause of cancer‐related death worldwide [1]. Despite advancements in diagnosis and treatment, prognosis remains unfavorable, with a 5‐year survival rate of only 38% in the United States [2]. Most patients are diagnosed with gastric cancer at an advanced stage, which significantly limits treatment options and contributes to worse outcomes [3, 4].

In 2022, the National Institute of Mental Health estimated that 23.1% of US adults (59.3 million) lived with a psychiatric disorder, including 6.0% (15.4 million) who had a serious psychiatric disorder. A serious psychiatric disorder is defined as a mental, behavioral, or emotional condition that causes significant functional impairment and interferes with one or more major life activity [5, 6]. Although prior literature has found that gastrointestinal cancer patients with comorbid psychiatric disorders have worse survival [7], the extent to which pre‐existing psychiatric disorders (PD), particularly serious pre‐existing psychiatric disorders (SPD), are associated with gastric cancer stage at diagnosis and survival remains poorly understood.

This study examines PD and SPD as independent risk factors for gastric cancer stage at diagnosis and survival. Based on prior evidence that PD is associated with poorer cancer outcomes [8], we hypothesized that patients with PD would be less likely to present with early (in situ/local) stage cancer and would have worse survival.

## 2 Materials and Methods

### 2.1 Data Source

Patients were identified using the Surveillance, Epidemiology, and End Results (SEER)–Medicare linked database. As of 2017, SEER covered approximately 34% of the national population [9]. The database includes detailed information on patient demographics, tumor characteristics (including stage, grade, and histology), cause of death, Medicare enrollment, and healthcare utilization. SEER‐Medicare is a widely used population‐based resource for cancer outcomes research.

### 2.2 Study Cohort

We queried the SEER‐Medicare database to identify patients aged 68 years and older diagnosed with gastric cancer between 2000 and 2017. Patients with a prior history of cancer (excluding non‐melanoma skin cancer) were excluded from the study. Eligible patients had continuous enrollment in Medicare Parts A and B, without Health Maintenance Organization (HMO) coverage, for at least 36 months prior to cancer diagnosis and a minimum of 6 months post diagnosis (or until death if they survived for less than 6 months) to ensure complete claims data. We excluded patients whose gastric cancer diagnosis was reported by death certificate or autopsy as well as those with missing or unknown information on diagnosis date, cancer stage, follow‐up, or death.

### 2.3 Identification of Psychiatric Disorders

Patients were classified as having PD if they had ≥ 1 inpatient or ≥ 2 outpatient Medicare claims associated with PD between 6 and 36 months prior to gastric cancer diagnosis. Claims were identified using International Classification of Diseases, Ninth Revision or Tenth Revision (ICD‐9, ICD‐10) codes (Table S1). The following conditions were included: depressive disorder, anxiety disorder, adjustment disorder, psychotic disorder, bipolar disorder, and schizophrenia.

Patients with a pre‐existing diagnosis of psychotic disorder, schizophrenia, bipolar disorder, or major depressive disorder (MDD) were classified as having SPD. While all psychiatric disorders represent a spectrum of severity, these conditions consistently demonstrate the highest burden of disease and disability [10, 11, 12]. However, the classification of MDD as SPD has been variable across studies due to its clinical heterogeneity [13, 14, 15]. Therefore, we conducted sensitivity analyses excluding patients with MDD from the SPD group to assess its impact on outcomes.

### 2.4 Survival Outcome/Endpoint Identification

Patients were followed from the date of gastric cancer diagnosis until the earliest of the following: death, discontinuation of Medicare Part A and B coverage, or the end of the study period (December 31, 2019). Survival outcomes—including death from gastric cancer, death from other causes, and survival through the study period—were determined using SEER data on vital status, survival time, and cause of death. Primary endpoints included cancer stage at diagnosis as well as survival outcomes, specifically overall mortality and gastric cancer‐specific mortality.

### 2.5 Treatment

A patient was considered to have received cancer treatment if they received one or more instances of chemotherapy, radiation, or surgery within 6 months of their cancer diagnosis. Receipt of treatment was extracted from Medicare claims using ICD‐9, ICD‐10, HCPCS (Healthcare Common Procedure Coding System), CPT (Current Procedural Terminology), and revenue center codes (Table S2).

### 2.6 Study Covariates

We obtained information about the following patient characteristics from the SEER record: age at cancer diagnosis, sex (male or female), race/ethnicity (non‐Hispanic White, non‐Hispanic Black, non‐Hispanic Asian/Pacific Islander, or Hispanic (all races)), rurality (urban or rural), marital status (married/partnered, single/unmarried, or unknown/other), and cancer grade (I, II, III, IV, or not determined). Histologic subtype was classified as adenocarcinoma ([ICD‐O‐3 codes: 8010, 8012, 8020, 8021, 8140–8145, 8200, 8201, 8210, 8211, 8221, 8230, 8255, 8260–8263, 8310, 8480, 8481, 8490, 8560, 8570‐8574, 8576]) or non‐adenocarcinoma. Anatomic site was grouped into cardia ([ICD‐O‐3 code C16.0]), non‐cardia ([C16.1–C16.6]), and overlapping/unknown ([C16.8–C16.9]). Cancer stage was classified as in situ, local, regional, or distant based on SEER summary staging.

Socioeconomic ranking was determined using county‐level indicators available in the SEER‐Medicare database, including education level, income, and poverty status [16]. Patients were assigned a score based on these socioeconomic attributes and categorized into quintiles ranging from 1 (lowest) to 5 (highest). Comorbidity burden was assessed using the Charlson comorbidity index [17], calculated from Medicare claims during the 12 months prior to gastric cancer diagnosis. Scores were grouped into four categories: 0, 1, 2, and ≥ 3.

### 2.7 Statistical Analysis

We conducted a series of analyses comparing the following exposure groups: PD versus NPD (no pre‐existing psychiatric disorders), SPD versus NSPD (non‐serious pre‐existing psychiatric disorders), and SPD versus NPD. We examined clinical and demographic variables between exposure groups using the Chi‐squared test for categorical variables and the Wilcoxon rank‐sum test for continuous, non‐normally distributed variables, such as age at cancer diagnosis.

We examined cancer stage at diagnosis using multinomial logistic regression and reported odds ratios (ORs) with 95% confidence intervals (CIs). We assessed the association between PD and mortality using Cox proportional hazards models for overall mortality and Fine‐Gray subdistribution hazard models for gastric cancer‐specific mortality to account for competing risks. We evaluated the proportional hazards assumption using Schoenfeld residuals and log–log plots. Models examining stage at diagnosis were adjusted for age at diagnosis, sex, race/ethnicity, rurality, marital status, socioeconomic quintile, and Charlson comorbidity index.

Mortality models included all covariates from the stage models, with additional adjustment for cancer stage, cancer grade, anatomic site, and histologic subtype. We reported hazard ratios (HRs) for all‐cause mortality and subdistribution hazard ratios (SHRs) for gastric cancer‐specific mortality, each with corresponding 95% CIs. Patients who remained alive at the end of the study period were censored. Follow‐up time was calculated from the date of cancer diagnosis until the earliest of death, loss to follow‐up, or end of the study period. All statistical tests were two‐sided, with significance defined as *p* < 0.05. Analyses were performed using SAS software (version 9.4, SAS Institute, Cary, NC) and R software (version 4.5.0, R Core Team 2025).

### 2.8 Sensitivity Analysis

We conducted several sensitivity analyses to assess the robustness of our findings. First, we restricted the cohort to patients with non‐cardia gastric adenocarcinoma, which represents the majority of gastric cancer cases [18, 19]. This restriction helped minimize heterogeneity in tumor biology related to anatomic site and histologic subtype. Second, to evaluate the effect of SPD classification, we performed a sensitivity analysis excluding patients with MDD from the SPD group, following a previously published analysis [20]. Lastly, to assess the impact of key covariates (e.g., cancer characteristics and treatment) on the association between PD and mortality, we ran the survival models sequentially adjusting for characteristic groups.

## 3 Results

### 3.1 Demographics and Cohort Characteristics

**FIGURE 1:**
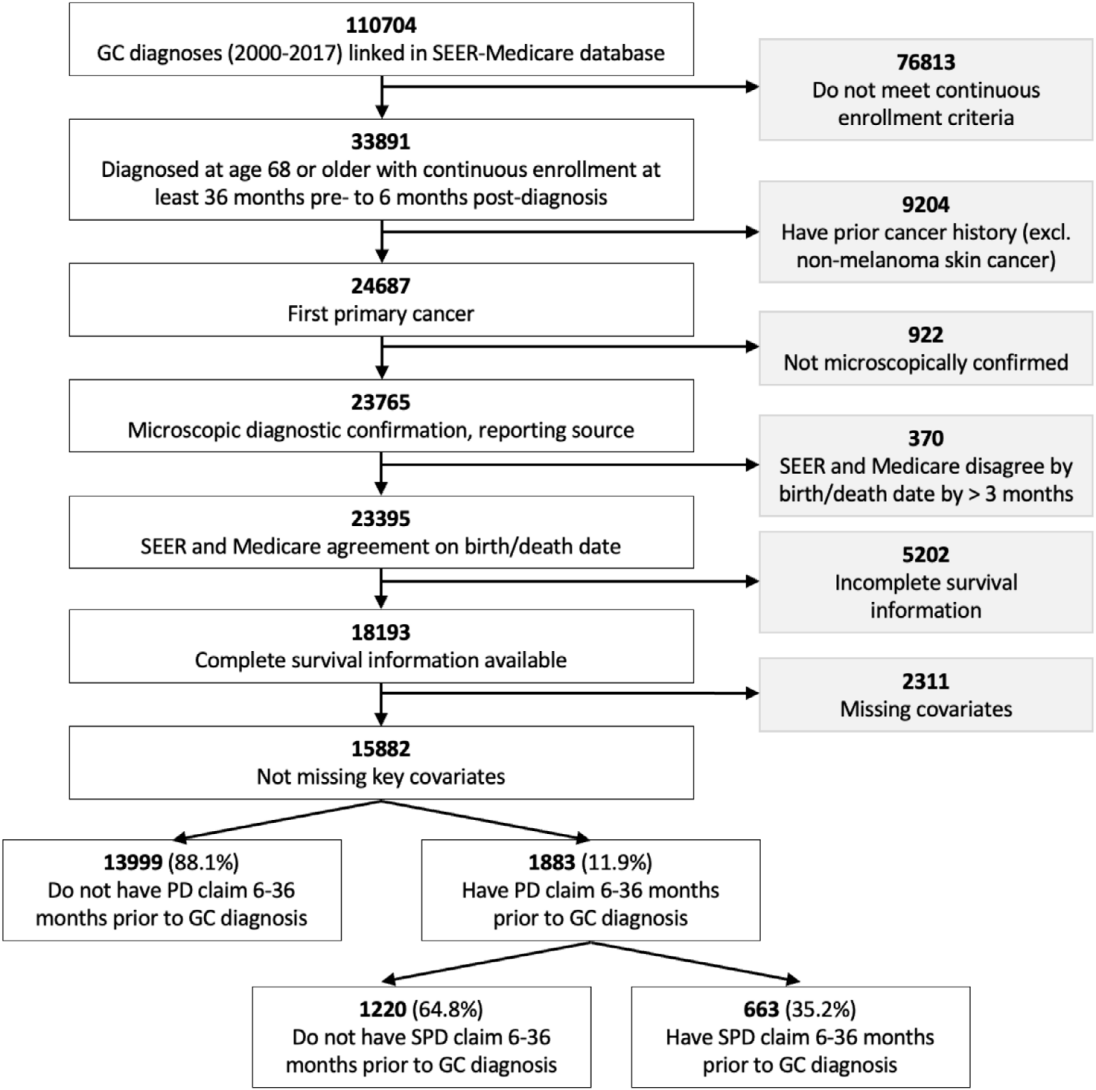
Patient cohort selection.

We identified 15,882 gastric cancer patients who met inclusion criteria, with a median age at diagnosis of 78 years (interquartile range [IQR], 73–83) (Figure 1). Overall, 1883 (11.9%) had at least one PD, and 663 had SPD (4.2% among all patients, or 35.2% among patients with PD). The median follow‐up (person‐time) was 9 (IQR, 2–32) months for PD patients and 11 (IQR, 3–37) months for NPD patients.

Compared with NPD patients, PD patients were more likely to be female (54.4% vs. 39.2%, *p* < 0.001) and White (77.0% vs. 70.4%, *p* < 0.001), more likely to be single or unmarried (53.1% vs. 39.5%, *p* < 0.001), more likely to be diagnosed with early‐stage cancer (45.1% vs. 35.6%, *p* < 0.001), more likely to have ≥ 3 comorbidities (56.9% vs. 42.0%, *p* < 0.001), and less likely to receive any form of cancer‐directed therapy (60.6% vs. 69.7%, *p* < 0.001). This pattern held across all treatment modalities, including chemotherapy (25.7% vs. 36.4%, *p* < 0.001), radiation therapy (18.2% vs. 23.7%, *p* < 0.001), and surgery (38.4% vs. 42.9%, *p* < 0.001). Among patients with PD, those classified as having SPD were more likely to have ≥ 3 comorbidities (63.7% vs. 53.3%, *p* < 0.001) and less likely to receive any form of cancer treatment (55.5% vs. 63.4%, *p* < 0.001) compared with NSPD patients (Table 1). Characteristics for patients where MDD is excluded as SPD are reported in Table S3.

**TABLE 1:** Clinical and demographic characteristics of gastric cancer patient cohort by PD and SPD Status.

Characteristic, *n* (%)	Total population (*N* = 15,882)	PD status			SPD status		
		PD (*N* = 1883)	NPD (*N* = 13,999)	*p*‐value	SPD (*N* = 663)	NSPD (*N* = 1220)	*p*‐value
Age at diagnosis [median years (IQR)]	78 (73, 83)	78 (73, 84)	78 (73, 83)	< 0.001	77 (73, 84)	78 (73, 84)	0.38
Sex				< 0.001			0.60
Male	9370 (59.0)	858 (45.6)	8512 (60.8)		308 (46.5)	550 (45.1)	
Female	6512 (41.0)	1025 (54.4)	5487 (39.2)		355 (53.5)	670 (54.9)	
Psychiatric disorder							
Depressive disorder (Excl. MDD)	816 (5.1)	816 (43.3)	—		197 (29.7)	619 (50.7)	
MDD	396 (2.5)	396 (21.0)	—		396 (59.7)	—	
Anxiety disorder	769 (4.8)	769 (40.8)	—		158 (23.8)	611 (50.1)	
Adjustment disorder	207 (1.3)	207 (11.0)	—		45 (6.8)	162 (13.3)	
Psychotic disorder	228 (1.4)	228 (12.1)	—		228 (34.4)	—	
Bipolar disorder	93 (0.6)	93 (4.9)	—		93 (14.0)	—	
Schizophrenia	59 (0.4)	59 (3.1)	—		59 (8.9)	—	
Race/ethnicity				< 0.001			0.09
Non‐hispanic white	11,308 (71.2)	1450 (77.0)	9858 (70.4)		508 (76.6)	942 (77.2)	
Non‐hispanic black	1659 (10.4)	175 (9.3)	1484 (10.6)		74 (11.2)	101 (8.3)	
Non‐hispanic asian/pacific islander	1630 (10.3)	114 (6.1)	1516 (10.8)		32 (4.8)	82 (6.7)	
Hispanic (all races)	1285 (8.1)	144 (7.6)	1141 (8.2)		49 (7.4)	95 (7.8)	
Marital status				< 0.001			0.11
Married/partnered	8688 (54.7)	796 (42.3)	7892 (56.4)		259 (39.1)	537 (44.0)	
Single/unmarried^a^	6524 (41.1)	999 (53.1)	5525 (39.5)		370 (55.8)	629 (51.6)	
Unknown/other	670 (4.2)	88 (4.7)	582 (4.2)		34 (5.1)	54 (4.4)	
Socioeconomic quintile				0.72			0.54
Q1‐lowest	3708 (23.3)	451 (24.0)	3257 (23.3)		166 (25.0)	285 (23.4)	
Q2	3479 (21.9)	401 (21.3)	3078 (22.0)		130 (19.6)	271 (22.2)	
Q3	2360 (14.9)	277 (14.7)	2083 (14.9)		104 (15.7)	173 (14.2)	
Q4	3818 (24.0)	469 (24.9)	3349 (23.9)		159 (24.0)	310 (25.4)	
Q5‐highest	2517 (15.8)	285 (15.1)	2232 (15.9)		104 (15.7)	181 (14.8)	
Rurality				0.94			0.008
Urban^b^	14,392 (90.6)	1705 (90.5)	12,687 (90.6)		617 (93.1)	1088 (89.2)	
Rural	1490 (9.4)	178 (9.5)	1312 (9.4)		46 (6.9)	132 (10.8)	
Charlson comorbidity index				< 0.001			< 0.001
0	3058 (19.3)	204 (10.8)	2854 (20.4)		54 (8.1)	150 (12.3)	
1	3019 (19.0)	294 (15.6)	2725 (19.5)		89 (13.4)	205 (16.8)	
2	2855 (18.0)	313 (16.6)	2542 (18.2)		98 (14.8)	215 (17.6)	
≥ 3	6950 (43.8)	1072 (56.9)	5878 (42.0)		422 (63.7)	650 (53.3)	
SEER cancer stage				< 0.001			0.70
In situ	236 (1.5)	43 (2.3)	193 (1.4)		12 (1.8)	31 (2.5)	
Local	5588 (35.2)	805 (42.8)	4783 (34.2)		289 (43.6)	516 (42.3)	
Regional	4867 (30.6)	479 (25.4)	4388 (31.3)		164 (24.7)	315 (25.8)	
Distant	5191 (32.7)	556 (29.5)	4635 (33.1)		198 (29.9)	358 (29.3)	
Cancer grade				< 0.001			0.79
I	1035 (6.5)	157 (8.3)	878 (6.3)		57 (8.6)	100 (8.2)	
II	3798 (23.9)	428 (22.7)	3370 (24.1)		157 (23.7)	271 (22.2)	
III	7396 (46.6)	809 (43.0)	6587 (47.1)		281 (42.4)	528 (43.3)	
IV	399 (2.5)	50 (2.7)	349 (2.5)		14 (2.1)	36 (3.0)	
Not determined, N/A	3254 (20.5)	439 (23.3)	2815 (20.1)		154 (23.2)	285 (23.4)	
Anatomic site				0.12			0.73
Non‐cardia	7563 (47.6)	934 (49.6)	6629 (47.4)		329 (49.6)	605 (49.6)	
Cardia	4955 (31.2)	552 (29.3)	4403 (31.5)		200 (30.2)	352 (28.9)	
Overlapping/unknown	3364 (21.2)	397 (21.1)	2967 (21.2)		134 (20.2)	263 (21.6)	
Histologic subtype				< 0.001			0.92
Adenocarcinoma	14,037 (88.4)	1604 (85.2)	12,433 (88.8)		566 (85.4)	1038 (85.1)	
Non‐adenocarcinoma	1845 (11.6)	279 (14.8)	1566 (11.2)		97 (14.6)	182 (14.9)	
Treatment				< 0.001			< 0.001
Any	10,905 (68.7)	1142 (60.6)	9763 (69.7)		368 (55.5)	774 (63.4)	
Chemotherapy	5583 (35.2)	484 (25.7)	5099 (36.4)		143 (21.6)	341 (28.0)	
Radiation	3653 (23.0)	342 (18.2)	3311 (23.7)		98 (14.8)	244 (20.0)	
Surgery	6728 (42.4)	724 (38.4)	6004 (42.9)		234 (35.3)	490 (40.2)	
None	4977 (31.3)	741 (39.4)	4236 (30.3)		295 (44.5)	446 (36.6)	

*Note: p*‐values indicate the statistical significance of differences between groups. *p*‐values less than 0.001 are reported as “< 0.001”. Some patients may have more than one PD; therefore, percentages may sum to more than 100%. Column percentages for characteristic groups may not sum to exactly 100% due to rounding.

Abbreviations: MDD, major depressive disorder; NPD, no pre‐existing psychiatric disorders; NSPD, non‐serious pre‐existing psychiatric disorders; PD, pre‐existing psychiatric disorders; SEER, Surveillance Epidemiology and End Results; SPD, serious pre‐existing psychiatric disorders.

^a^ Includes single, separated, divorced, and widowed patients.

^b^ Includes large metropolitan counties and adjacent urban counties.

Among 1883 patients with PD, 513 (27.2%) had multiple PD diagnoses. Specifically, 373 (19.8%) had 2 diagnoses, 98 (5.2%) had 3 diagnoses, and 42 (2.2%) had > 3 PD diagnoses. Schizophrenia and bipolar disorder were most frequently associated with multiple PD diagnoses: 67.8% of patients with schizophrenia and 72.0% of patients with bipolar disorder had multiple PD diagnoses. Overall, patients with PD had a median of 1 (IQR, 1–2) PD, while those with SPD had a median of 2 (IQR, 1–2) PD.

### 3.2 Cancer Stage

PD patients had significantly higher odds of being diagnosed with early‐stage rather than distant‐stage gastric cancer (aOR: 1.26, 95% CI [1.12, 1.42]) (Table 2). This association remained when the analysis was restricted to non‐cardia adenocarcinoma (aOR: 1.40, 95% CI [1.16, 1.70]) (Table S4). Among individual PD diagnoses, the association was statistically significant only for patients with depressive disorder (excluding MDD) (aOR: 1.27, 95% CI [1.07, 1.50]), anxiety disorder (aOR: 1.33, 95% CI [1.11, 1.58]), and adjustment disorder (aOR: 1.43, 95% CI [1.03, 2.00]) (Table S5). Among racial/ethnic groups, both non‐Hispanic White and non‐Hispanic Asian/Pacific Islander patients with PD had increased odds of being diagnosed with early‐stage cancer ((aOR: 1.27, 95% CI [1.11, 1.45]) and (aOR: 2.25, 95% CI [1.28, 3.95]), respectively) (Table S6). However, there was no difference observed in cancer stage at diagnosis between SPD and NSPD patients regardless of whether MDD was included in the SPD classification (Tables 2 and S7).

**TABLE 2:** Odds of in situ/local or regional stage cancer diagnosis vs. distant stage cancer diagnosis: Comparisons by PD vs. NPD, SPD vs. NSPD, and SPD vs. NPD.

Group	Stage at diagnosis	OR (95% CI)	aOR^a^ (95% CI)
*PD* vs. *NPD (ref.)*			
	In situ/Local	1.42 (1.27, 1.59)*	1.26 (1.12, 1.42)*
	Regional	0.91 (0.80, 1.04)	0.91 (0.80, 1.04)
	Distant	Ref.	Ref.
*SPD* vs. *NSPD (ref.)*			
	In situ/Local Regional Distant	1.00 (0.80, 1.24) 0.94 (0.73, 1.22) Ref.	0.97 (0.77, 1.22) 0.92 (0.71, 1.20) Ref.
*SPD* vs. *NPD (ref.)*			
	In situ/Local Regional Distant	1.42 (1.18, 1.70) * 0.87 (0.71, 1.08) Ref.	1.22 (1.01, 1.47) * 0.87 (0.70, 1.08) Ref.

Abbreviations: CI, confidence interval; NPD, no pre‐existing psychiatric disorders; NSPD, non‐serious pre‐existing psychiatric disorders; OR, odds ratio; PD, pre‐existing psychiatric disorders; SPD, serious pre‐existing psychiatric disorders.

^a^ Models adjust for age at diagnosis, sex, race/ethnicity, rurality, marital status, socioeconomic quintile, and Charlson comorbidity index.

* Represents a statistically significant result.

### 3.3 Mortality

There were 1539 (81.7%) overall deaths among PD patients and 11,580 (82.7%) among NPD patients. There were 1136 (60.3%) gastric cancer‐specific deaths among PD patients and 9004 (64.3%) among NPD patients. Compared with NPD patients, those with PD had an increased hazard of both overall mortality (aHR: 1.17, 95% CI [1.11, 1.23]) and cancer‐specific mortality (aSHR: 1.13, 95% CI [1.07, 1.21]) (Table 3). These associations remained consistent when the analysis was restricted to patients with non‐cardia adenocarcinoma (Table S8).

**TABLE 3:** Hazard ratios for overall and gastric cancer‐specific mortality: Comparisons of PD vs. NPD, SPD vs. NSPD, and SPD vs. NPD.

Group	HR/SHR (95% CI)	aHR^a^/aSHR^a^ (95% CI)
*PD* vs. *NPD (ref.)*		
Overall Mortality	1.07 (1.02, 1.13)*	1.17 (1.11, 1.23)*
Cancer‐Specific Mortality	1.00 (0.94, 1.06)	1.13 (1.07, 1.21)*
*SPD* vs. *NSPD (ref.)*		
Overall Mortality	1.18 (1.06, 1.31)*	1.23 (1.10, 1.37)*
Cancer‐Specific Mortality	1.12 (1.00, 1.26)	1.16 (1.04, 1.30)*
*SPD* vs. *NPD (ref.)*		
Overall Mortality	1.19 (1.10, 1.30)*	1.33 (1.22, 1.45)*
Cancer‐Specific Mortality	1.08 (0.97, 1.19)	1.25 (1.14, 1.38)*

Abbreviations: CI, confidence interval; HR, hazard ratio; NPD, no pre‐existing psychiatric disorders; NSPD, non‐serious pre‐existing psychiatric disorders; PD, pre‐existing psychiatric disorders; SHR, subdistribution hazard ratio; SPD, serious pre‐existing psychiatric disorders.

^a^ Models adjust for age at diagnosis, sex, race/ethnicity, rurality, marital status, socioeconomic quintile, Charlson comorbidity index, cancer stage, cancer grade, anatomic site, and histologic subtype. Overall mortality hazard ratios (HR) were calculated using the Cox proportional hazards model, while cancer‐specific subdistribution hazard ratios (SHR) were calculated using the Fine‐Gray subdistribution hazard model.

* Represents a statistically significant result.

Patients with SPD also had elevated risks of overall mortality (aHR: 1.33, 95% CI [1.22, 1.45]) and gastric cancer–specific mortality (aSHR: 1.25, 95% CI [1.14, 1.38]) compared with NPD patients (Table 3). When MDD was excluded from the SPD definition, the associations were more pronounced: overall mortality (aHR: 1.58, 95% CI [1.40, 1.77]) and cancer‐specific mortality (aSHR: 1.36, 95% CI [1.18, 1.56]) (Table S9). Survival differences by cancer stage were minimal and similar to the overall results, but patients with PD and regional‐stage cancer faced higher hazards of both overall mortality (aHR: 1.28, 95% CI [1.16, 1.42]) and cancer‐specific mortality (aSHR: 1.26, 95% CI [1.12, 1.42]) compared with NPD patients (Table S10).

When stratified by individual PD, a higher risk of overall mortality was observed in patients with schizophrenia and psychotic disorder compared with NPD patients ((aHR: 1.77, 95% CI [1.35, 2.32]) and (aHR: 1.61, 95% CI [1.41, 1.85]), respectively). A higher risk of cancer‐specific mortality was also observed in patients with schizophrenia and psychotic disorder compared with NPD patients ((aSHR: 1.43, 95% CI [1.03, 1.98]) and (aSHR: 1.39, 95% CI [1.17, 1.64]), respectively) (Table S11).

Sensitivity analyses with sequential covariate adjustment showed that accounting for cancer characteristics increased hazard ratios, while adjustment for treatment attenuated the association between PD and mortality (Table S12).

## 4 Discussion

In this retrospective cohort study, we sought to determine the relationship between PD and gastric cancer outcomes. We found that patients with PD were diagnosed with earlier stage cancer, opposite to our initial hypothesis, yet they experienced significantly worse survival outcomes than those without PD. Although prior studies have suggested an association between PD and advanced stage cancer diagnosis [8, 21], other studies such as Chang et al. (2014) [22] did not observe this association, highlighting the need for further investigation in gastric cancer. Earlier stage at diagnosis among patients with PD may reflect increased healthcare engagement due to frequent interactions with providers for psychiatric and medical comorbidities. For example, Sporinova et al. (2019) found that adults with chronic diseases and comorbid mental health disorders have higher healthcare utilization and associated costs than those without mental health disorders [23]. Increased healthcare contact may promote early cancer detection through opportunistic evaluation or incidental findings.

Furthermore, we found that earlier gastric cancer diagnosis did not translate into improved survival. PD has been associated with reduced adherence to guideline‐concordant care for gastric cancer [24] and replicated for breast cancer [25, 26], highlighting PD as a key variable that influences patient treatment and thus cancer outcomes. In our cohort, patients with PD had a higher comorbidity burden and were less likely to receive cancer‐directed treatments, including surgery, chemotherapy, and radiation compared with NPD patients. Adjusting for treatment in our survival models partially attenuated the association of PD with worse survival (Table S12). However, survival differences by cancer stage were minimal and similar to the overall results (Table S10), suggesting that worse survival in the PD group was not driven by differences in stage at diagnosis.

Survival disparities in PD patients may reflect system‐level barriers, such as fragmented care [27, 28], limited care access [29, 30], and limited social support [31, 32]. They may also result from patient‐level factors, including lower treatment uptake due to financial and time constraints [33, 34, 35, 36, 37] or challenges adhering to complex regimens [38, 39]. Psychiatric disorders may also influence cancer progression through direct biological pathways; for example, depression and other psychiatric conditions have been linked to dysregulation of the hypothalamic‐pituitary‐adrenal (HPA) axis [40] and dysregulated cortisol excretion in response to acute stressors [41].

In our cohort, not only were medical comorbidities magnified in patients with SPD, but we also found that many SPD patients had multiple psychiatric diagnoses. The poorer survival observed in patients with SPD highlights the heterogeneity of psychiatric disorders and suggests that certain subgroups may be particularly vulnerable, warranting targeted investigation.

Consistent with our findings, numerous population‐based studies have reported worse cancer outcomes among patients with PD across multiple malignancies, including breast, colon, prostate, pancreatic, and lung cancer [20, 42, 43, 44, 45]. Specific to gastrointestinal cancers, Harris et al. (2020) conducted a SEER‐Medicare analysis demonstrating that patients with comorbid psychiatric disorders have lower overall and cancer‐specific survival [7]. A major difference in the Harris et al. (2020) study, however, is the definition of PD exposure: the analysis included diagnoses made from 12 months before to 6 months after cancer diagnosis, potentially capturing psychiatric conditions arising in response to gastric cancer. In contrast, we focused on psychiatric disorders diagnosed 6 months to 3 years prior to cancer diagnosis to capture pre‐existing psychiatric comorbidity. With our updated SEER gastric cancer cohort, we demonstrate a higher cancer‐specific and overall mortality for gastric cancer patients with PD and SPD versus NPD, while adjusting for stage at diagnosis and accounting for competing risks.

The American Society for Clinical Oncology recommends screening for anxiety and depression regularly throughout the trajectory of cancer care and emphasizes consideration of a patient's psychiatric history when selecting treatment approaches [46]. We note that these recommendations do not address identification of potentially more serious psychopathologies (such as schizophrenia and psychotic disorder); thus, we emphasize the importance of a more comprehensive evaluation for PD at the time of cancer diagnosis.

## 5 Limitations

A key strength of this study is the use of the SEER‐Medicare database, which offers a large, nationally representative cohort of older adults with detailed clinical and demographic data. Our approach is well‐established in population‐based research using claims data to identify patients with psychiatric conditions. However, several limitations must be acknowledged. The cohort is restricted to Medicare beneficiaries aged ≥ 68 years without prior cancers. Thus, the findings may not be fully generalizable to younger gastric cancer patients, those with non‐Medicare insurance, or individuals with prior cancers. Psychiatric disorder rates may also differ in these excluded groups. Additionally, prior research has raised concerns about the sensitivity and specificity of ICD codes for identifying psychiatric disorders, particularly for conditions not requiring hospitalization or ongoing treatment, and for their inability to capture variation in symptom severity. Davis et al. (2016) [47] found that claims‐based identification of psychotic disorder, schizophrenia, bipolar disorder, and depression is more reliable (positive predictive value > 75%) compared with anxiety disorders (< 60%). Consistent with these limitations, reliance on administrative data in our observational study may lead to underestimation of psychiatric disorder prevalence and precludes causal inference. Lastly, administrative data does not fully capture factors that may affect outcomes in this patient population (such as psychiatric disorder treatment [45, 48] and substance abuse [49, 50]). Given the observational design of our study, residual confounding cannot be ruled out and may contribute to the observed associations.

## 6 Conclusion

Our findings highlight critical disparities in gastric cancer outcomes for PD patients, with particularly poor survival among those with SPD. Despite being diagnosed with earlier stage cancer, patients with PD experienced worse survival. Further characterization and mitigation of the complex barriers that these patients face is urgently needed. While interventions to enhance access to care, improve psychiatric‐oncology coordination, and promote guideline‐concordant treatment among PD patients may hold promise, understanding the mechanisms underlying cancer outcome disparities is essential. Future research should clarify these drivers to develop effective targeted interventions.

## Author Contributions

**Josephine Soddano:** conceptualization (lead), data curation (lead), formal analysis (lead), investigation (lead), methodology (lead), writing – original draft (lead). **Sophie Wagner:** data curation (lead), formal analysis (equal), methodology (equal), writing – original draft (equal). **Ji Yoon Yoon:** formal analysis (equal), methodology (equal), writing – original draft (equal). **Jeong Yun Yang:** methodology (equal), writing – original draft (equal). **Ling Chen:** methodology (supporting), writing – review and editing (equal). **Yongmei Huang:** methodology (supporting), writing – review and editing (equal). **Sheila D. Rustgi:** writing – review and editing (equal). **Yoanna S. Pumpalova:** writing – review and editing (supporting). **Jennifer S. Ferris:** conceptualization (equal), formal analysis (equal), methodology (equal), project administration (lead), supervision (lead), writing – original draft (equal). **Chin Hur:** conceptualization (equal), funding acquisition (lead), methodology (equal), project administration (lead), supervision (lead), writing – original draft (equal).

## Funding

Chin Hur receives research and salary support through National Cancer Institute (NCI) U01CA265729. Jeong Yun Yang receives research and salary support through National Institute of Diabetes and Digestive and Kidney Diseases (NIDDK) T32DK083256‐16. Ji Yoon Yoon receives research and salary support through National Institutes of Health (NIH) T32CA225617 and American Cancer Society (ACS) CSDG‐24‐1319963‐01‐ESED. The content of this manuscript is solely the responsibility of the authors and does not necessarily represent the official views of the NIH. The funders had no role in the design and conduct of the study; collection, management, analysis, and interpretation of the data; preparation, review, or approval of the manuscript; and decision to submit the manuscript for publication.

## Ethics Statement

This study utilized publicly available, de‐identified data from the Surveillance, Epidemiology, and End Results (SEER)‐Medicare database. The Columbia University Institutional Review Board determined this study to be exempt.

## Conflicts of Interest

The authors declare no conflicts of interest.

## Supporting information


**Data S1:** Supporting Information.

## Data Availability

We will not be making data available to the public in concordance with SEER‐Medicare regulations. The datasets used to conduct this study are available upon approval of a research protocol from the National Cancer Institute. Instructions for obtaining these data are available at https://healthcaredelivery.cancer.gov/seermedicare/obtain/.
