# Supplementary material for: The Impact of Pre‐Existing Psychiatric Disorders on Gastric Cancer Stage and Mortality in Older Adults
Source: Cancer Med. 2026 Jun 3;15(6):e71622. doi: 10.1002/cam4.71622 (PMC13239988; doi:10.1002/cam4.71622)
Supplement: Supplementary file 1 — Data S1: Supporting Information. [file CAM4-15-e71622-s001.docx]

**Supplemental Materials**

| **Supplemental Table 1.** Psychiatric Disorder Diagnosis Codes Used for Query.  **Supplemental Table 2.** Treatment Codes Used for Query.  **Supplemental Table 3.** Clinical and Demographic Characteristics of Gastric Cancer Patient Cohort where MDD is Excluded as SPD.  **Supplemental Table 4.** Odds of In situ/Local or Regional Stage Cancer Diagnosis vs. Distant Stage Cancer Diagnosis in Patients with Non-cardia Gastric Adenocarcinoma: Comparisons by PD vs. NPD, SPD vs. NSPD, and SPD vs. NPD.  **Supplemental Table 5.** Odds of In situ/Local or Regional Stage Cancer Diagnosis vs. Distant Stage Cancer Diagnosis: Comparisons of Individual PD vs. NPD.  **Supplemental Table 6.** Odds of In situ/Local or Regional Stage Cancer Diagnosis vs. Distant Stage Cancer Diagnosis in Patients with PD vs. NPD, Stratified by Characteristic Subgroup.  **Supplemental Table 7.** Odds of In situ/Local or Regional Stage Cancer Diagnosis vs. Distant Stage Cancer Diagnosis: Comparisons of SPD vs. NSPD and SPD vs. NPD, Excluding MDD from SPD Classification.  **Supplemental Table 8.** Hazard Ratios for Overall and Gastric Cancer-Specific Mortality in Patients with Non-cardia Gastric Adenocarcinoma: Comparisons by PD vs. NPD, SPD vs. NSPD, and SPD vs. NPD.  **Supplemental Table 9.** Hazard Ratios for Overall and Gastric Cancer-Specific Mortality: Comparisons of SPD vs. NSPD and SPD vs. NPD, Excluding MDD from SPD Classification.  **Supplemental Table 10.** Hazard Ratios for Overall and Gastric Cancer-Specific Mortality in Patients with PD, Stratified by Characteristic Subgroup.  **Supplemental Table 11.** Hazard Ratios for Overall and Gastric Cancer-Specific Mortality: Comparisons of Individual PD vs. NPD.  **Supplemental Table 12.** Hazard Ratios for Overall and Gastric Cancer-Specific Mortality: Comparisons of PD vs. NPD, SPD vs. NSPD, and SPD vs. NPD with Sequential Adjustment of Covariate Groups. |
| --- |

| **Supplemental Table 1.** Psychiatric Disorder Diagnosis Codes Used for Query. | | |
| --- | --- | --- |
| **Psychiatric Disorder Query** | **Code Set** | **Codes** |
| Anxiety Disorder | ICD-9-CM | 293.84, 300-300.09, 300.3, 308-308.9, 309.81 |
|  | ICD-10-CM | F06.4, F41-F41.9, F42-F42.9, F43.0, F43.10-F43.12 |
| Depressive Disorder (Excl. MDD) | ICD-9-CM | 300.4, 311 |
|  | ICD-10-CM | F34.1 |
| MDD | ICD-9-CM | 296.2-296.36 |
|  | ICD-10-CM | F32.0-F32.5, F32.9, F33-F33.9 |
| Bipolar Disorder | ICD-9-CM | 296.0-296.16, 296.4-296.99 |
|  | ICD-10-CM | F30-F30.9, F31-F31.9 |
| Schizophrenia | ICD-9-CM | 295-295.95 |
|  | ICD-10-CM | F20-F20.9, F21, F25-F25.9 |
| Psychotic Disorder | ICD-9-CM | 293.81, 293.82, 297-298.9 |
|  | ICD-10-CM | F22, F23, F24, F28, F29, F06.0, F06.2 |
| Adjustment Disorder | ICD-9-CM | 309 (excl. 309.81) |
|  | ICD-10-CM | F43.2-F43.29 |

**Note.** *Abbreviations:* ICD-9-CM, International Classification of Diseases, Ninth Revision, Clinical

Modification; ICD-10-CM, International Classification of Diseases, Tenth Revision, Clinical Modification; HCPCS, Healthcare Common Procedure Coding System; MDD, Major Depressive Disorder. Codes used to identify psychiatric disorders were informed by prior literature^1,2^.

| **Supplemental Table 2.** Treatment Codes Used for Query. | | |
| --- | --- | --- |
| **Treatment Query** | **Code Set** | **Codes** |
| Chemotherapy | ICD-9-CM Diagnosis | V58.1, V66.2, V67.2 |
|  | ICD-9-CM Procedure | 99.25 |
|  | ICD-10-CM | Z51.1 |
|  | ICD-10-PCS | 3E03305, 3E04305 |
|  | HCPCS | C1166, C1167, C1178, C9110, C9205, C9207, C9213-C9216, C9411, C9414-C9419, C942x, C9430-C9438, G0359-G0362, J7150, J85xx-J87xx, J8999, J9xxx, Q0083-Q0085 |
|  | CPT | 964xx, 9651x-9654x |
|  | Revenue Center | 0331, 0332, 0335 |
| Radiation | ICD-9-CM Diagnosis | V58.0, V66.1, V67.1 |
|  | ICD-9-CM Procedure | 92.21-92.29 |
|  | ICD-10-CM | Z51.0 |
|  | ICD-10-PCS | Dxxxxxx |
|  | CPT | 77401-77499, 77520, 77523, 77750-77799 |
|  | Revenue Center | 0330, 0333 |
| Gastric Surgery | ICD-9-CM Procedure | 43.42, 43.49, 43.5, 43.6, 43.7, 43.8, 43.81, 43.89, 43.9, 43.91, 43.99 |
|  | ICD-10-PCS | 0DB4[0-8]ZZ, 0DT4[0-8]ZZ, 0DB6[0-8]ZZ, 0DT6[0-8]ZZ, 0DB7[0-8]ZZ, 0DT7[0-8]ZZ |
|  | CPT | 43620-43622, 43631-43634 |

**Note.** *Abbreviations:* ICD-9-CM, International Classification of Diseases, Ninth Revision, Clinical

Modification; ICD-10-CM, International Classification of Diseases, Tenth Revision, Clinical Modification; ICD-10-PCS, International Classification of Diseases, Tenth Revision, Procedure Coding System; HCPCS, Healthcare Common Procedure Coding System; CPT, Current Procedural Terminology. Codes used to identify treatment were informed by prior literature^3^.

| **Supplemental Table 3.** Clinical and Demographic Characteristics of Gastric Cancer Patient Cohort where MDD is Excluded as SPD. | | | | | | | | | | |  |
| --- | --- | --- | --- | --- | --- | --- | --- | --- | --- | --- | --- |
|  | |  |  |  | **PD Status** | | | **SPD Status** | | | |
|  | |  | Characteristic, n (%) | Total population | PD | NPD | p-value | SPD | NSPD | p-value | |
|  |  |  |  | (N=15882) | (N=1883) | (N=13999) |  | (N=329) | (N=1554) |  |  |
| Age at diagnosis [median years (IQR)] | | | | 78 (73, 83) | 78 (73, 84) | 78 (73, 83) | <0.001 | 78 (73, 84) | 78 (73, 84) | 0.31 | |
| Sex | | | |  |  |  | <0.001 |  |  | 0.30 | |
|  | Male | | | 9370 (59.0) | 858 (45.6) | 8512 (60.8) |  | 159 (48.3) | 699 (45.0) |  | |
|  | Female | | | 6512 (41.0) | 1025 (54.4) | 5487 (39.2) |  | 170 (51.7) | 855 (55.0) |  | |
|  | Psychiatric Disorder | | |  |  |  |  |  |  |  | |
|  | Depressive Disorder (Excl. MDD) | | | 816 (5.1) | 816 (43.3) | - |  | 86 (26.1) | 730 (47.0) |  | |
|  | MDD | | | 396 (2.5) | 396 (21.0) | - |  | 62 (18.8) | 334 (21.5) |  | |
|  | Anxiety Disorder | | | 769 (4.8) | 769 (40.8) | - |  | 69 (21.0) | 700 (45.0) |  | |
|  | Adjustment Disorder | | | 207 (1.3) | 207 (11.0) | - |  | 19 (5.8) | 188 (12.1) |  | |
|  | Psychotic Disorder | | | 228 (1.4) | 228 (12.1) | - |  | 228 (69.3) | - |  | |
|  | Bipolar Disorder | | | 93 (0.6) | 93 (4.9) | - |  | 93 (28.3) | - |  | |
|  | Schizophrenia | | | 59 (0.4) | 59 (3.1) | - |  | 59 (17.9) | - |  | |
| Race/Ethnicity | | | |  |  |  | <0.001 |  |  | <0.001 | |
|  | | Non-Hispanic White | | 11308 (71.2) | 1450 (77.0) | 9858 (70.4) |  | 244 (74.2) | 1206 (77.6) |  | |
|  | | Non-Hispanic Black | | 1659 (10.4) | 175 (9.3) | 1484 (10.6) |  | 50 (15.2) | 125 (8.0) |  | |
|  | | Non-Hispanic Asian/Pacific Islander | | 1630 (10.3) | 114 (6.1) | 1516 (10.8) |  | 13 (4.0) | 101 (6.5) |  | |
|  | | Hispanic (All Races) | | 1285 (8.1) | 144 (7.6) | 1141 (8.2) |  | 22 (6.7) | 122 (7.9) |  | |
| Marital Status | | | |  |  |  | <0.001 |  |  | 0.08 | |
|  | | Married/Partnered | | 8688 (54.7) | 796 (42.3) | 7892 (56.4) |  | 121 (36.8) | 675 (43.4) |  | |
|  | | Single/Unmarried^a^ | | 6524 (41.1) | 999 (53.1) | 5525 (39.5) |  | 191 (58.1) | 808 (52.0) |  | |
|  | | Unknown/Other | | 670 (4.2) | 88 (4.7) | 582 (4.2) |  | 17 (5.2) | 71 (4.6) |  | |
| Socioeconomic Quintile | | | |  |  |  | 0.72 |  |  | 0.05 | |
|  | | Q1-lowest | | 3708 (23.3) | 451 (24.0) | 3257 (23.3) |  | 94 (28.6) | 357 (23.0) |  | |
|  | | Q2 | | 3479 (21.9) | 401 (21.3) | 3078 (22.0) |  | 63 (19.1) | 338 (21.8) |  | |
|  | | Q3 | | 2360 (14.9) | 277 (14.7) | 2083 (14.9) |  | 58 (17.6) | 219 (14.1) |  | |
|  | | Q4 | | 3818 (24.0) | 469 (24.9) | 3349 (23.9) |  | 71 (21.6) | 398 (25.6) |  | |
|  | | Q5-highest | | 2517 (15.8) | 285 (15.1) | 2232 (15.9) |  | 43 (13.1) | 242 (15.6) |  | |
|  | | | |  |  |  |  |  |  |  | |
| Rurality | | | |  |  |  | 0.94 |  |  | 0.46 | |
|  | | Urban^b^ | | 14392 (90.6) | 1705 (90.5) | 12687 (90.6) |  | 302 (91.8) | 1403 (90.3) |  | |
|  | | Rural | | 1490 (9.4) | 178 (9.5) | 1312 (9.4) |  | 27 (8.2) | 151 (9.7) |  | |
| Charlson Comorbidity Index | | | |  |  |  | <0.001 |  |  | <0.001 | |
|  | | 0 | | 3058 (19.3) | 204 (10.8) | 2854 (20.4) |  | 24 (7.3) | 180 (11.6) |  | |
|  | | 1 | | 3019 (19.0) | 294 (15.6) | 2725 (19.5) |  | 39 (11.9) | 255 (16.4) |  | |
|  | | 2 | | 2855 (18.0) | 313 (16.6) | 2542 (18.2) |  | 41 (12.5) | 272 (17.5) |  | |
|  | | ≥3 | | 6950 (43.8) | 1072 (56.9) | 5878 (42.0) |  | 225 (68.4) | 847 (54.5) |  | |
| SEER Cancer Stage | | | |  |  |  | <0.001 |  |  | 0.82 | |
|  | | In situ | | 236 (1.5) | 43 (2.3) | 193 (1.4) |  | * | 34 (2.2) |  | |
|  | | Local | | 5588 (35.2) | 805 (42.8) | 4783 (34.2) |  | * | 663 (42.7) |  | |
|  | | Regional | | 4867 (30.6) | 479 (25.4) | 4388 (31.3) |  | 78 (23.7) | 401 (25.8) |  | |
|  | | Distant | | 5191 (32.7) | 556 (29.5) | 4635 (33.1) |  | 100 (30.4) | 456 (29.3) |  | |
| Cancer Grade | | | |  |  |  | <0.001 |  |  | 0.52 | |
|  | | I | | 1035 (6.5) | 157 (8.3) | 878 (6.3) |  | 35 (10.6) | 122 (7.9) |  | |
|  | | II | | 3798 (23.9) | 428 (22.7) | 3370 (24.1) |  | 71 (21.6) | 357 (23.0) |  | |
|  | | III | | 7396 (46.6) | 809 (43.0) | 6587 (47.1) |  | 139 (42.2) | 670 (43.1) |  | |
|  | | IV | | 399 (2.5) | 50 (2.7) | 349 (2.5) |  | * | 43 (2.8) |  | |
|  | | Not determined, N/A | | 3254 (20.5) | 439 (23.3) | 2815 (20.1) |  | * | 362 (23.3) |  | |
| Anatomic Site | | | |  |  |  | 0.12 |  |  | 0.84 | |
|  | | Non-cardia | | 7563 (47.6) | 934 (49.6) | 6629 (47.4) |  | 166 (50.5) | 768 (49.4) |  | |
|  | | Cardia | | 4955 (31.2) | 552 (29.3) | 4403 (31.5) |  | 92 (28.0) | 460 (29.6) |  | |
|  | | Overlapping/Unknown | | 3364 (21.2) | 397 (21.1) | 2967 (21.2) |  | 71 (21.6) | 326 (21.0) |  | |
| Histologic Subtype | | | |  |  |  | <0.001 |  |  | 0.29 | |
|  | | Adenocarcinoma | | 14037 (88.4) | 1604 (85.2) | 12433 (88.8) |  | 287 (87.2) | 1317 (84.7) |  | |
|  | | Non-adenocarcinoma | | 1845 (11.6) | 279 (14.8) | 1566 (11.2) |  | 42 (12.8) | 237 (15.3) |  | |
|  | | Treatment | |  |  |  | <0.001 |  |  | <0.001 | |
|  | | Any | | 10905 (68.7) | 1142 (60.6) | 9763 (69.7) |  | 172 (52.3) | 970 (62.4) |  | |
|  | | Chemotherapy | | 5583 (35.2) | 484 (25.7) | 5099 (36.4) |  | 54 (16.4) | 430 (27.7) |  | |
|  | | Radiation | | 3653 (23.0) | 342 (18.2) | 3311 (23.7) |  | 46 (14.0) | 296 (19.0) |  | |
|  | | Surgery | | 6728 (42.4) | 724 (38.4) | 6004 (42.9) |  | 116 (35.3) | 608 (39.1) |  | |
|  | | None | | 4977 (31.3) | 741 (39.4) | 4236 (30.3) |  | 157 (47.7) | 584 (37.6) |  | |

**Note***. Abbreviations:* PD, pre-existing psychiatric disorders; SPD, serious pre-existing psychiatric disorders; NPD, no pre-existing psychiatric disorders; NSPD, non-serious pre-existing psychiatric disorders; MDD, major depressive disorder; SEER, Surveillance Epidemiology and End Results. p-values indicate the statistical significance of differences between groups. p-values less than 0.001 are reported as “<0.001”. Some patients may have more than one PD; therefore, percentages may sum to more than 100%. Column percentages for characteristic groups may not sum to exactly 100% due to rounding.

^a^ Includes single, separated, divorced, and widowed patients.

^b^ Includes large metropolitan counties and adjacent urban counties.

* Omitted to comply with National Cancer Institute (NCI) Reporting Guidelines.

**Supplemental Table 4.** Odds of In situ/Local or Regional Stage Cancer Diagnosis vs. Distant Stage Cancer Diagnosis in Patients with Non-cardia Gastric Adenocarcinoma: Comparisons by PD vs. NPD, SPD vs. NSPD, and SPD vs. NPD.

| **Group** | **Stage at Diagnosis** | **OR (95% CI)** | **aOR**^a^ **(95% CI)** |
| --- | --- | --- | --- |
| *PD vs. NPD (ref.)* | | | |
|  | In situ/Local | 1.56 (1.30, 1.89) * | 1.40 (1.16, 1.70) * |
|  | Regional | 1.08 (0.88, 1.32) | 1.06 (0.87, 1.30) |
|  | Distant | Ref. | Ref. |
| *SPD vs. NSPD (ref.)* | | |  |
|  | In situ/Local  Regional  Distant | 0.89 (0.61, 1.28)  0.85 (0.57, 1.26)  Ref. | 0.86 (0.59, 1.26)  0.83 (0.55, 1.26)  Ref. |
| *SPD vs. NPD (ref.)* |  |  |  |
|  | In situ/Local  Regional  Distant | 1.45 (1.07, 1.95) *  0.97 (0.70, 1.34)  Ref. | 1.27 (0.94, 1.72)  0.95 (0.68, 1.31)  Ref. |
| **Note.** *Abbreviations:* PD, pre-existing psychiatric disorders; SPD, serious pre-existing psychiatric disorders; NPD, no pre-existing psychiatric disorders; NSPD, non-serious pre-existing psychiatric disorders; OR, odds ratio; CI, confidence interval.  ^a^ Models adjust for age at diagnosis, sex, race/ethnicity, rurality, marital status, socioeconomic quintile, and Charlson comorbidity index.  * Represents a statistically significant result. | | | |

**Supplemental Table 5.** Odds of In situ/Local or Regional Stage Cancer Diagnosis vs. Distant Stage Cancer Diagnosis: Comparisons of Individual PD vs. NPD.

| **Group vs. NPD (ref.)** | **n**^a^ | **Stage at Diagnosis** | **OR (95% CI)** | **aOR**^b^ **(95% CI)** |  |
| --- | --- | --- | --- | --- | --- |
| *Depressive Disorder* 816  *(Excl. MDD)* | | | | |  |
|  |  | In situ/Local | 1.41 (1.20, 1.67) * | 1.27 (1.07, 1.50) * |  |
|  |  | Regional | 0.87 (0.72, 1.06) | 0.88 (0.72, 1.06) |  |
|  |  | Distant | Ref. | Ref. |  |
| *MDD* 396 | | | | |  |
|  |  | In situ/Local  Regional  Distant | 1.43 (1.13, 1.81) *  0.93 (0.71, 1.22)  Ref. | 1.25 (0.98, 1.59)  0.93 (0.71, 1.21)  Ref. |  |
| *Anxiety Disorder* | 769 |  |  |  |  |
|  |  | In situ/Local  Regional  Distant | 1.49 (1.25, 1.77) *  0.95 (0.78, 1.15)  Ref. | 1.33 (1.11, 1.58) *  0.95 (0.78, 1.16)  Ref. |  |
| *Adjustment Disorder* | 207 |  |  |  |  |
|  |  | In situ/Local  Regional  Distant | 1.62 (1.17, 2.25) *  0.95 (0.65, 1.38)  Ref. | 1.43 (1.03, 2.00) *  0.94 (0.64, 1.38)  Ref. |  |
| *Psychotic Disorder* | 228 |  |  |  |  |
|  |  | In situ/Local  Regional  Distant | 1.43 (1.05, 1.94) *  0.81 (0.57, 1.16)  Ref. | 1.14 (0.83, 1.55)  0.80 (0.56, 1.15)  Ref. |  |
| *Bipolar Disorder* | 93 |  |  |  |  |
|  |  | In situ/Local  Regional  Distant | 1.23 (0.77, 1.97)  0.72 (0.41, 1.25)  Ref. | 1.14 (0.71, 1.83)  0.72 (0.41, 1.26)  Ref. |  |
| *Schizophrenia* | 59 |  |  |  |  |
|  |  | In situ/Local  Regional  Distant | 1.64 (0.91, 2.98)  0.75 (0.36, 1.56)  Ref. | 1.44 (0.79, 2.63)  0.73 (0.35, 1.53)  Ref. |  |
| **Note**. *Abbreviations:* PD, pre-existing psychiatric disorders; NPD, no pre-existing psychiatric disorders; MDD, major depressive disorder; OR, odds ratio; CI, confidence interval.  ^a^ n refers to the number of individuals with each PD condition.  ^b^ Models adjust for age at diagnosis, sex, race/ethnicity, rurality, marital status, socioeconomic quintile, and Charlson comorbidity index.  * Represents a statistically significant result. | | | | |  |

| **Supplemental Table 6.** Odds of In situ/Local or Regional Stage Cancer Diagnosis vs. Distant Stage Cancer Diagnosis in Patients with PD vs. NPD, Stratified by Characteristic Subgroup. | | | |
| --- | --- | --- | --- |
| **PD vs. NPD (ref.)** | **Stage at Diagnosis** | **OR (95% CI)** | **aOR**^a^ **(95% CI)** |
| *Sex* |  |  |  |
| Male | In situ/Local vs. Distant (*ref*.) | 1.33 (1.13, 1.58) * | 1.23 (1.04, 1.46) * |
|  | Regional vs. Distant (*ref*.) | 0.97 (0.81, 1.16) | 0.96 (0.80, 1.16) |
| Female | In situ/Local vs. Distant (*ref*.) | 1.39 (1.18, 1.62) * | 1.28 (1.09, 1.50) * |
|  | Regional vs. Distant (*ref*.) | 0.86 (0.71, 1.04) | 0.86 (0.71, 1.04) |
| *Age at diagnosis* | |  |  |
| ≤ 75 years | In situ/Local vs. Distant (*ref*.) | 1.32 (1.10, 1.58) * | 1.17 (0.97, 1.42) |
|  | Regional vs. Distant (*ref*.) | 0.88 (0.72, 1.07) | 0.89 (0.73, 1.10) |
| > 75 years | In situ/Local vs. Distant (*ref*.) | 1.48 (1.28, 1.72) * | 1.34 (1.15, 1.55) * |
|  | Regional vs. Distant (*ref*.) | 0.93 (0.79, 1.10) | 0.93 (0.79, 1.10) |
| *Race/Ethnicity* |  |  |  |
| NH Black, NH AAPI, and    Hispanic (All Races) | In situ/Local vs. Distant (*ref*.)  Regional vs. Distant (*ref*.) | 1.50 (1.18, 1.91) *  1.02 (0.78, 1.34) | 1.22 (0.95, 1.56)  0.99 (0.75, 1.30) |
| NH White | In situ/Local vs. Distant (*ref*.)  Regional vs. Distant (*ref*.) | 1.41 (1.24, 1.61) *  0.88 (0.76, 1.03) | 1.27 (1.11, 1.45) *  0.88 (0.76, 1.02) |
| NH Black | In situ/Local vs. Distant (*ref*.)  Regional vs. Distant (*ref*.) | 1.46 (1.00, 2.13)  1.08 (0.72, 1.64) | 1.08 (0.73, 1.59)  0.96 (0.63, 1.47) |
| NH AAPI | In situ/Local vs. Distant (*ref*.)  Regional vs. Distant (*ref*.) | 2.50 (1.44, 4.33) *  1.61 (0.89, 2.91) | 2.25 (1.28, 3.95) *  1.76 (0.96, 3.22) |
| Hispanic (All Races) | In situ/Local vs. Distant (*ref*.)  Regional vs. Distant (*ref*.) | 1.29 (0.86, 1.92)  0.84 (0.53, 1.33) | 0.96 (0.63, 1.47)  0.79 (0.49, 1.27) |
| *Charlson Comorbidity Index* | |  |  |
| 0 | In situ/Local vs. Distant (*ref*.) | 1.69 (1.21, 2.36) * | 1.66 (1.18, 2.33) * |
|  | Regional vs. Distant (*ref*.) | 0.95 (0.66, 1.37) | 1.00 (0.69, 1.45) |
| 1-2 | In situ/Local vs. Distant (*ref*.) | 1.22 (1.00, 1.49) | 1.20 (0.98, 1.46) |
|  | Regional vs. Distant (*ref*.) | 0.84 (0.68, 1.05) | 0.87 (0.70, 1.08) |
| ≥ 3 | In situ/Local vs. Distant (*ref*.) | 1.27 (1.09, 1.49) * | 1.23 (1.05, 1.44) * |
|  | Regional vs. Distant (*ref*.) | 0.90 (0.75, 1.08) | 0.92 (0.76, 1.10) |
| *Non-cardia adenocarcinoma cases, only* | |  |  |
|  | In situ/Local vs. Distant (*ref*.) | 1.56 (1.30, 1.89) * | 1.40 (1.16, 1.70) * |
|  | Regional vs. Distant (*ref*.) | 1.08 (0.88, 1.32) | 1.06 (0.87, 1.30) |

**Note**. Abbreviations: PD, pre-existing psychiatric disorders; NPD, no pre-existing psychiatric disorders; OR, odds ratio; CI, confidence interval; NH, non-Hispanic; AAPI, Asian American/Pacific Islander.

^a^ Models adjust for age at diagnosis, sex, race/ethnicity, rurality, marital status, socioeconomic quintile, and Charlson comorbidity index. Variables used for stratification were not included as covariates in corresponding models.

* Represents a statistically significant result.

**Supplemental Table 7.** Odds of In situ/Local or Regional Stage Cancer Diagnosis vs. Distant Stage Cancer Diagnosis: Comparisons of SPD vs. NSPD and SPD vs. NPD, Excluding MDD from SPD Classification.

| **Group** | **Stage at Diagnosis** | **OR (95% CI)** | **aOR**^a^ **(95% CI)** |
| --- | --- | --- | --- |
| *SPD vs. NSPD (ref.)* | | |  |
|  | In situ/Local  Regional  Distant | 0.99 (0.75, 1.31)  0.89 (0.64, 1.23)  Ref. | 0.95 (0.71, 1.26)  0.86 (0.62, 1.20)  Ref. |
| *SPD vs. NPD (ref.)* |  |  |  |
|  | In situ/Local  Regional  Distant | 1.41 (1.09, 1.82) *  0.82 (0.61, 1.11)  Ref. | 1.19 (0.91, 1.54)  0.82 (0.61, 1.10)  Ref. |
| **Note.** *Abbreviations:* SPD, serious pre-existing psychiatric disorders; NPD, no pre-existing psychiatric disorders; NSPD, non-serious pre-existing psychiatric disorders; MDD, major depressive disorder; OR, odds ratio; CI, confidence interval.  ^a^ Models adjust for age at diagnosis, sex, race/ethnicity, rurality, marital status, socioeconomic quintile, and Charlson comorbidity index.  * Represents a statistically significant result. | | | |

**Supplemental Table 8.** Hazard Ratios for Overall and Gastric Cancer-Specific Mortality in Patients with Non-cardia Gastric Adenocarcinoma: Comparisons by PD vs. NPD, SPD vs. NSPD, and SPD vs. NPD.

|  | | |
| --- | --- | --- |
| **Group** | **HR/SHR (95% CI)** | **aHR**^a^**/aSHR**^a^ **(95% CI)** |
| *PD vs. NPD (ref.)* | | |
| Overall Mortality | 1.11 (1.02, 1.20) * | 1.16 (1.07, 1.27) * |
| Cancer-Specific Mortality | 1.04 (0.95, 1.14) | 1.14 (1.03, 1.25) * |
| *SPD vs. NSPD (ref.)* |  |  |
| Overall Mortality  Cancer-Specific Mortality | 1.17 (0.99, 1.37)  1.07 (0.89, 1.29) | 1.21 (1.02, 1.42) *  1.09 (0.91, 1.30) |
| *SPD vs. NPD (ref.)* |  |  |
| Overall Mortality  Cancer-Specific Mortality | 1.22 (1.07, 1.39) *  1.09 (0.93, 1.27) | 1.30 (1.14, 1.48) *  1.20 (1.03, 1.40) * |
| **Note.** *Abbreviations:* PD, pre-existing psychiatric disorders; SPD, serious pre-existing psychiatric disorders; NPD, no pre-existing psychiatric disorders; NSPD, non-serious pre-existing psychiatric disorders; HR, hazard ratio; SHR, subdistribution hazard ratio; CI, confidence interval. Overall mortality hazard ratios (HR) were calculated using the Cox proportional hazards model, while cancer-specific subdistribution hazard ratios (SHR) were calculated using the Fine-Gray subdistribution hazard model.  ^a^ Models adjust for age at diagnosis, sex, race/ethnicity, rurality, marital status, socioeconomic quintile, Charlson comorbidity index, cancer stage, and cancer grade.  * Represents a statistically significant result. | | |

**Supplemental Table 9.** Hazard Ratios for Overall and Gastric Cancer-Specific Mortality: Comparisons of SPD vs. NSPD and SPD vs. NPD, Excluding MDD from SPD Classification.

|  | | |
| --- | --- | --- |
| **Group** | **HR/SHR (95% CI)** | **aHR**^a^**/aSHR**^a^ **(95% CI)** |
| *SPD vs. NSPD (ref.)* |  |  |
| Overall Mortality  Cancer-Specific Mortality | 1.40 (1.23, 1.59) *  1.19 (1.02, 1.39) * | 1.42 (1.24, 1.62) *  1.23 (1.07, 1.42) * |
| *SPD vs. NPD (ref.)* |  |  |
| Overall Mortality  Cancer-Specific Mortality | 1.43 (1.27, 1.60) *  1.17 (1.01, 1.35) * | 1.58 (1.40, 1.77) *  1.36 (1.18, 1.56) * |
| **Note.** *Abbreviations:* SPD, serious pre-existing psychiatric disorders; NPD, no pre-existing psychiatric disorders; NSPD, non-serious pre-existing psychiatric disorders; MDD, major depressive disorder; HR, hazard ratio; SHR, subdistribution hazard ratio; CI, confidence interval. Overall mortality hazard ratios (HR) were calculated using the Cox proportional hazards model, while cancer-specific subdistribution hazard ratios (SHR) were calculated using the Fine-Gray subdistribution hazard model.  ^a^ Models adjust for age at diagnosis, sex, race/ethnicity, rurality, marital status, socioeconomic quintile, Charlson comorbidity index, cancer stage, cancer grade, anatomic site, and histologic subtype.  * Represents a statistically significant result. | | |

| **Supplemental Table 10.** Hazard Ratios for Overall and Gastric Cancer-Specific Mortality in Patients with PD, Stratified by Characteristic Subgroup. | | | | | | |
| --- | --- | --- | --- | --- | --- | --- |
|  | | | **Overall Mortality** | | **Gastric Cancer-Specific Mortality** | |
| **PD vs. NPD (ref.)** | | | **HR (95% CI)** | **aHR**^a^ **(95% CI)** | **SHR (95% CI)** | **aSHR**^a^ **(95% CI)** |
| *Total Cohort* | | | 1.07 (1.02, 1.13) * | 1.17 (1.11, 1.23) * | 1.00 (0.94, 1.06) | 1.13 (1.07, 1.21) * |
| *Age at Diagnosis* | | |  |  |  |  |
|  | ≤ 75 years | | 1.07 (0.98, 1.17) | 1.15 (1.05, 1.26) * | 0.97 (0.88, 1.08) | 1.08 (0.97, 1.20) |
|  | > 75 years | | 1.07 (1.00, 1.14) | 1.17 (1.09, 1.25) * | 1.00 (0.93, 1.08) | 1.15 (1.07, 1.24) * |
| *Sex* | | |  |  |  |  |
|  | Male | | 1.15 (1.07, 1.24) * | 1.15 (1.07, 1.25) * | 1.07 (0.98, 1.17) | 1.13 (1.04, 1.23) * |
|  | Female | | 1.04 (0.97, 1.13) | 1.18 (1.09, 1.27) * | 0.97 (0.89, 1.06) | 1.15 (1.05, 1.25) * |
| *Stage* | | |  |  |  |  |
|  | In situ/Local | | 1.18 (1.08, 1.29) * | 1.15 (1.05, 1.26) * | 1.08 (0.95, 1.21) | 1.09 (0.96, 1.23) |
|  | Regional | | 1.29 (1.17, 1.43) * | 1.28 (1.16, 1.42) * | 1.25 (1.11, 1.41) * | 1.26 (1.12, 1.42) * |
| Distant | | | 1.09 (1.00, 1.20) | 1.11 (1.01, 1.21) * | 1.07 (0.98, 1.18) | 1.10 (1.00, 1.20) |
| *Race/Ethnicity* | | |  |  |  |  |
|  | NH Black, NH AAPI, and    Hispanic (All Races) | | 1.03 (0.92, 1.15) | 1.14 (1.02, 1.28) * | 0.94 (0.83, 1.07) | 1.07 (0.94, 1.23) |
|  | NH White | | 1.08 (1.01, 1.15) * | 1.18 (1.11, 1.26) * | 1.00 (0.94, 1.08) | 1.15 (1.08, 1.24) * |
|  | NH Black | | 1.12 (0.94, 1.32) | 1.25 (1.05, 1.49) * | 0.98 (0.80, 1.20) | 1.13 (0.93, 1.39) |
|  | NH AAPI | | 0.85 (0.67, 1.07) | 1.04 (0.82, 1.31) | 0.78 (0.59, 1.03) | 0.97 (0.71, 1.32) |
|  | Hispanic (All Races) | | 1.01 (0.83, 1.23) | 1.07 (0.88, 1.31) | 0.95 (0.76, 1.18) | 1.03 (0.83, 1.27) |
| *Charlson Comorbidity Index* | | |  |  |  |  |
|  | 0 | | 0.95 (0.81, 1.12) | 1.17 (0.99, 1.38) | 0.95 (0.80, 1.13) | 1.18 (0.98, 1.41) |
|  | 1-2 | | 1.02 (0.93, 1.13) | 1.11 (1.01, 1.22) * | 0.99 (0.89, 1.10) | 1.09 (0.98, 1.21) |
|  | ≥ 3 | | 1.12 (1.04, 1.20) * | 1.22 (1.13, 1.31) * | 1.04 (0.95, 1.13) | 1.15 (1.06, 1.25) * |
| *Anatomic Site* | | |  |  |  |  |
|  | Cardia | | 1.16 (1.06, 1.28) * | 1.16 (1.06, 1.28) * | 1.14 (1.02, 1.26) * | 1.19 (1.07, 1.33) * |
|  | Non-cardia | | 1.05 (0.97, 1.14) | 1.16 (1.07, 1.25) * | 0.97 (0.89, 1.06) | 1.13 (1.03, 1.24) * |
|  |  | Non-cardia adenocarcinoma | 1.11 (1.02, 1.20) * | 1.16 (1.07, 1.27) * | 1.04 (0.95, 1.14) | 1.14 (1.03, 1.25) * |
| *Treatment Status* | | |  |  |  |  |
|  | Treatment | | 1.04 (0.97, 1.12) | 1.15 (1.07, 1.23) * | 0.95 (0.88, 1.03) | 1.08 (1.00, 1.18) |
|  | No Treatment | | 0.99 (0.91, 1.08) | 1.05 (0.96, 1.14) | 0.94 (0.86, 1.03) | 1.06 (0.97, 1.16) |
| **Note.** *Abbreviations:* PD, pre-existing psychiatric disorders; NPD, no pre-existing psychiatric disorders; HR, hazard ratio; SHR, subdistribution hazard ratio; CI, confidence interval; NH, non-Hispanic; AAPI, Asian American/Pacific Islander. Overall mortality hazard ratios (HR) were calculated using the Cox proportional hazards model, while cancer-specific subdistribution hazard ratios (SHR) were calculated using the Fine-Gray subdistribution hazard model. Variables used for stratification were not included as covariates in the corresponding models.  ^a^ Models adjust for age at diagnosis, sex, race/ethnicity, rurality, marital status, socioeconomic quintile, Charlson comorbidity index, cancer stage, cancer grade, anatomic site, and histologic subtype.  * Represents a statistically significant result. | | | | | | |

**Supplemental Table 11.** Hazard Ratios for Overall and Gastric Cancer-Specific Mortality: Comparisons of Individual PD vs. NPD.

| **Group vs. NPD (ref.)** | **n**^a^ | **HR/SHR (95% CI)** | **aHR**^b^**/aSHR**^b^ **(95% CI)** |  |  |
| --- | --- | --- | --- | --- | --- |
| *Depressive Disorder*  *(Excl. MDD)* | 816 | | |  |  |
| Overall Mortality |  | 1.07 (1.00, 1.17) | 1.18 (1.09, 1.28) * |  |  |
| Cancer-Specific Mortality |  | 0.99 (0.91, 1.09) | 1.13 (1.03, 1.23) * |  |  |
| *MDD* | 396 |  |  |  |  |
| Overall Mortality  Cancer-Specific Mortality |  | 1.03 (0.92, 1.15)  0.98 (0.86, 1.12) | 1.16 (1.04, 1.30) *  1.14 (1.00, 1.29) |  |  |
| *Anxiety Disorder* | 769 |  |  |  |  |
| Overall Mortality  Cancer-Specific Mortality |  | 0.98 (0.90, 1.06)  0.92 (0.84, 1.02) | 1.08 (0.99, 1.17)  1.06 (0.96, 1.16) |  |  |
| *Adjustment Disorder* | 207 |  |  |  |  |
| Overall Mortality  Cancer-Specific Mortality |  | 1.03 (0.88, 1.20)  0.97 (0.80, 1.17) | 1.12 (0.96, 1.31)  1.17 (0.96, 1.42) |  |  |
| *Psychotic Disorder* | 228 |  |  |  |  |
| Overall Mortality  Cancer-Specific Mortality |  | 1.55 (1.35, 1.77) *  1.22 (1.02, 1.46) * | 1.61 (1.41, 1.85) *  1.39 (1.17, 1.64) * |  |  |
| *Bipolar Disorder* | 93 |  |  |  |  |
| Overall Mortality  Cancer-Specific Mortality |  | 1.19 (0.95, 1.49)  1.04 (0.79, 1.36) | 1.37 (1.09, 1.72) *  1.19 (0.92, 1.54) |  |  |
| *Schizophrenia* | 59 |  |  |  |  |
| Overall Mortality  Cancer-Specific Mortality |  | 1.44 (1.11, 1.89) *  1.10 (0.78, 1.56) | 1.77 (1.35, 2.32) *  1.43 (1.03, 1.98) * |  |  |
| **Note.** *Abbreviations:* PD, pre-existing psychiatric disorders; NPD, no pre-existing psychiatric disorders; MDD, major depressive disorder; HR, hazard ratio; SHR, subdistribution hazard ratio; CI, confidence interval. Overall mortality hazard ratios (HR) were calculated using the Cox proportional hazards model, while cancer-specific subdistribution hazard ratios (SHR) were calculated using the Fine-Gray subdistribution hazard model.  ^a^ n refers to the number of individuals with each PD condition.  ^b^ Models adjust for age at diagnosis, sex, race/ethnicity, rurality, marital status, socioeconomic quintile, Charlson comorbidity index, cancer stage, cancer grade, anatomic site, and histologic subtype.  * Represents a statistically significant result. | | | |  |  |

**Supplemental Table 12.** Hazard Ratios for Overall and Gastric Cancer-Specific Mortality: Comparisons of PD vs. NPD, SPD vs. NSPD, and SPD vs. NPD with Sequential Adjustment of Covariate Groups.

|  | **Unadjusted Model** | |  | | **Adjusted Models** | |  | |  |  |
| --- | --- | --- | --- | --- | --- | --- | --- | --- | --- | --- |
|  |  | | **Model 1**^a^ | | **Model 2**^b^ | | **Model 3**^c^ | | **Model 4**^d^ |  |
| **Group** | | **HR/SHR (95% CI)** | | **aHR/aSHR**  **(95% CI)** | | **aHR/aSHR**  **(95% CI)** | | **aHR/aSHR**  **(95% CI)** | **aHR/aSHR**  **(95% CI)** |  |
| *PD vs. NPD (ref.)* | |  | |  | |  | |  |  |  |
| Overall Mortality | | 1.07 (1.02, 1.13) * | | 1.06 (1.00, 1.12) | | 1.11 (1.05, 1.17) * | | 1.17 (1.11, 1.23) * | 1.12 (1.06, 1.18) * |  |
| Cancer Mortality | | 1.00 (0.94, 1.06) | | 1.02 (0.96, 1.08) | | 1.06 (0.99, 1.13) | | 1.13 (1.07, 1.21) * | 1.08 (1.01, 1.15) * |  |
| *SPD vs. NSPD (ref.)* | | | |  | |  | |  |  |  |
| Overall Mortality | | 1.18 (1.06, 1.31) * | | 1.18 (1.06, 1.32) * | | 1.23 (1.10, 1.36) * | | 1.23 (1.10, 1.37) * | 1.20 (1.08, 1.34) * |  |
| Cancer Mortality | | 1.12 (1.00, 1.26) | | 1.16 (1.03, 1.31) * | | 1.19 (1.05, 1.34) * | | 1.16 (1.04, 1.30) * | 1.13 (1.01, 1.27) * |  |
| *SPD vs. NPD (ref.)* | | | |  | |  | |  |  |  |
| Overall Mortality  Cancer Mortality | | 1.19 (1.10, 1.30) *  1.08 (0.97, 1.19) | | 1.18 (1.09, 1.29) *  1.12 (1.01, 1.25) * | | 1.27 (1.17, 1.38) *  1.19 (1.07, 1.31) * | | 1.33 (1.22, 1.45) *  1.25 (1.14, 1.38) * | 1.25 (1.15, 1.37) *  1.17 (1.06, 1.28) * |  |

**Note.** *Abbreviations:* PD, pre-existing psychiatric disorders; SPD, serious pre-existing psychiatric disorders; NPD, no pre-existing psychiatric disorders; NSPD, non-serious pre-existing psychiatric disorders; HR, hazard ratio; SHR, subdistribution hazard ratio; CI, confidence interval. Overall mortality hazard ratios (HR) were calculated using the Cox proportional hazards model, while cancer-specific subdistribution hazard ratios (SHR) were calculated using the Fine-Gray subdistribution hazard model.

^a^ Model 1 adjusts for age at diagnosis, sex, race/ethnicity, rurality, marital status, socioeconomic quintile, and Charlson comorbidity index.

^b^ Model 2 includes all Model 1 covariates, plus cancer grade, anatomic site, and histologic subtype.

^c^ Model 3 includes all Model 2 covariates, plus cancer stage.

^d^ Model 4 includes all Model 3 covariates, plus treatment.

* Represents a statistically significant result.

**References**

1. Harris JP, Kashyap M, Humphreys JN, Pollom EL, Chang DT. The clinical and financial cost of mental disorders among elderly patients with gastrointestinal malignancies. Cancer Med. 2020;9(23):8912-8922. doi:10.1002/cam4.3509

2. Paredes AZ, Hyer JM, Tsilimigras DI, et al. Association of pre-existing mental illness with all-cause and cancer-specific mortality among Medicare beneficiaries with pancreatic cancer. HPB. 2021;23(3):451-458. doi:10.1016/j.hpb.2020.08.002

3. Hoffman KE, Neville BA, Mamon HJ, et al. Adjuvant therapy for elderly patients with resected gastric adenocarcinoma: population-based practices and treatment effectiveness. Cancer. 2012;118(1):248-257. doi:10.1002/cncr.26248
